# Early photoperiodic cues modulate capacity for adult cold tolerance in the yellow fever mosquito *Aedes aegypti*

**DOI:** 10.1242/jeb.252460

**Published:** 2026-06-18

**Authors:** Ella H. M. De Nicola, Marshall W. Ritchie, Daniel A. Hahn, Kyle K. Biggar, Heath A. MacMillan

**Affiliations:** ^1^ Department of Biology, Carleton University, Ottawa, Ontario, K1S 5B6, Canada; ^2^ Department of Entomology & Nematology, University of Florida, Gainesville, FL 32611, USA; ^3^ Institute of Biochemistry, Carleton University, Ottawa, Ontario, K1S 5B6, Canada

**Keywords:** *Aedes aegypti*, Seasonal plasticity, Cold acclimation, Photoperiod

## Abstract

Climate change is driving the rapid range expansion of *Aedes aegypti* into temperate regions, presenting novel seasonal cues that can affect seasonal plasticity. Seasonal plasticity in *Ae. aegypti* is well studied in eggs but remains understudied in adults. Here, we investigated the effects of photoperiod and temperature on thermal plasticity, measured by the ability to cold acclimate, and other dormancy-related traits in female *Ae. aegypti* adults. Cold acclimation was done under a short-day (SD) and long-day (LD) photoperiod. LD photoperiod was introduced at three life stages: parental generation, egg state and adult acclimation, to determine if timing of LD photoperiod introduction affected thermal plasticity. LD photoperiod had a significant influence on both cold tolerance metrics assessed, chill coma onset temperature and survival after a cold stress. Introduction of LD photoperiod reduced thermal plasticity, with the magnitude of change in plasticity depending on when LD was introduced. At warm temperatures, LD photoperiod modified the rhythmic expression profile of *timeless*, one of the circadian clock genes analysed in this study, but not the other, *period*. In contrast, cold acclimation abolished the cyclical expression of both clock genes. Cold acclimation under both photoperiods suppressed blood-feeding behaviour, which resumed upon warming, but cold acclimation did not change relative transcript abundance of dormancy-implicated genes. This study presents the first evidence of an adult photosensitive seasonal phenotype in *Ae. aegypti*, with improved cold tolerance and reproductive quiescence. These findings contribute to a deeper understanding of how environmental cues may facilitate the continued expansion of this important disease vector into temperate environments.

## Teaser

**Highlighted Article:** Short photoperiods improve cold tolerance and reproductive quiescence in *Aedes aegypti* adults, potentially facilitating its movement into temperate climates.

## INTRODUCTION

The yellow fever mosquito, *Aedes aegypti* (Diptera: Culicidae) is a vector of several important arboviral diseases including dengue fever, Zika, Chikungunya and yellow fever (Jentes et al., 2011; Simmons et al., 2012; Powell and Tabachnick, 2013; Souza-Neto et al., 2019; Kraemer et al., 2019). Collectively, these diseases cause close to one million deaths annually and place substantial pressure on global health systems, disproportionately impacting low-income countries (World Health Organization, 2020). *Aedes aegypti* is a tropical species, with historical origins in the Southwest Indian Ocean region (Soghigian et al., 2020) and was considered to be limited to tropical and subtropical regions (Kramer et al., 2020). However, this species is currently undergoing rapid range expansion, largely driven by climate change and urbanization, and has now established populations on every continent except Antarctica (Kraemer et al., 2019; Giordano et al., 2020; Rubio et al., 2020; Johnston et al., 2025; Lozano et al., 2025). As *Ae. aegypti* spreads more into poleward, temperate climates, this species has and will encounter cooler conditions than those experienced in its native habitats which imposes new physiological challenges.

The geographic distribution of a species, and capacity to expand its range, is closely related to its critical thermal limits, defined as the temperature boundaries within which an organism can maintain physiological performance and survival (Kellermann et al., 2012; Overgaard et al., 2014; MacLean et al., 2016; Dickens et al., 2018; van Heerwaarden and Sgrò, 2021; Couper et al., 2024). As most insects are ectotherms and cannot internally regulate their body temperature, environmental temperatures directly influence body temperature and physiological function. Low temperature exposure can elicit various forms of phenotypic plasticity that enhance cold tolerance, ranging from rapid cellular responses to developmental or seasonal responses (Chown and Terblanche, 2006; Sgrò et al., 2026). One of the most well-studied forms of thermal plasticity in insects is cold acclimation, which involves physiological adjustments following prolonged exposure to a sublethal temperature typically over days or weeks (Hazell and Bale, 2011; Andersen et al., 2017; Yerushalmi et al., 2018; Rohr et al., 2018; Enriquez et al., 2018; Jass et al., 2019; Bayley et al., 2020). Cold acclimation improves survival at low temperatures through mechanisms such as enhanced ionic and osmotic homeostasis, lipid membrane remodelling and regulation of metabolism (Koštál et al., 2006; Teets and Denlinger, 2013; MacMillan et al., 2015b). These changes can occur within hours to days, allowing insects to respond to short-term changes in temperature and are generally reversible when favourable conditions return (Slotsbo et al., 2016).

In addition to acclimation, many insects survive adverse environmental conditions by entering states of dormancy (Denlinger, 2023). Diapause is a hormonally regulated, preprogrammed state of dormancy that occurs at a single life stage and allows insects to avoid an unfavourable season. By contrast, quiescence is an environmentally induced response that can occur at multiple life stages and is rapidly reversible when favourable conditions return (Denlinger, 2023). Both of these states of dormancy are strongly associated with an improved ability to survive environmental stress, including cold exposure (Diniz et al., 2017).

Although the capacity of *Ae. aegypti* to express low temperature plasticity is not fully characterized, we know that this species is capable of cold acclimation (Jass et al., 2019). Survival following a chronic cold exposure was significantly improved through cold acclimation in both larvae and adult *Ae. aegypti*. Along with improved low temperature tolerance, cold-acclimated females showed a reluctance to blood feed at 15°C (Jass et al., 2019), both hallmarks of dormancy in other mosquito species (Denlinger and Armbruster, 2014). No diapause has been definitively described in *Ae. Aegypti*; however, the egg, embryo and pharate larvae can all enter quiescence (Sota and Mogi, 1992; da Silva and da Silva, 1999; Rezende et al., 2008; McMeniman and O'Neill, 2010; Perez and Noriega, 2012, 2013; Vargas et al., 2014; Prasad et al., 2023). Whether a cold acclimation phenotype is representative of a more complex adult *Ae. aegypti* dormancy phenotype, such as quiescence or diapause, is unknown, leaving a significant gap in our understanding of the mechanisms underlying continued range expansion of the species into cooler climates.

Our understanding of mosquito dormancy, specifically diapause, comes primarily from studies of adult diapause in the common house mosquito, *Culex pipiens*, an important vector of West Nile virus (Denlinger and Armbruster, 2014). Adult diapause in mosquitoes is primarily characterized by an increase in cold tolerance (Rinehart et al., 2006), fat accumulation and a behaviour switch from seeking a blood meal to feeding on sugar (Robich and Denlinger, 2005; Denlinger and Armbruster, 2014). These phenotypic changes are regulated by the juvenile hormone (JH) and insulin signaling (IS) pathways, which converge to control the activity of the transcription factor FOXO through the neuropeptides allatotropin and insulin-like peptide 1 (ILP-1), respectively (Spielman, 1974; Sim and Denlinger, 2008, 2009, 2013; Denlinger and Armbruster, 2014; Kang et al., 2014; Sim et al., 2015). Under short photoperiods and low temperatures, the IS and JH pathways are suppressed, resulting in FOXO activation and the expression of genes that result in the diapause phenotype, including reproductive arrest (Sim et al., 2015). Thus, changes in endocrine signalling are critical to the environmental induction of dormancy (Denlinger, 2023).

Environmental cues, such as photoperiod and temperature can regulate the entry into and exit out of seasonal phenotypes via endocrine signalling (Denlinger and Armbruster, 2014; Denlinger, 2023). Both temperature and photoperiod interact to regulate seasonal phenotypes in insects, but photoperiod is often a dominant cue because it is generally a more reliable signal for seasonal timing owing to its predictability relative to temperature (Denlinger et al., 2017). Photoperiod thresholds for seasonal responses are often quantified and compared using the critical photoperiod, the photoperiod at which half of a population enters a dormant physiological state, arresting development (Denlinger, 1972; Denlinger and Armbruster, 2014). The photoperiodic programming of seasonal phenotypes in mosquitoes can occur across several different life stages, including via maternal effects, where the photoperiod experienced by the mother influences dormancy expression in her offspring (Mousseau and Fox, 1998; Denlinger, 2023).

Photoperiodic time measurement is processed through the circadian clock, which has long been implicated in seasonal time measurement (Dolezel, 2015; Saunders, 2020). The circadian clock consists of a transcriptional negative feedback loop in which core clock genes *period* (PER) and *timeless* (TIM) form a heterodimer that suppresses activity of transcription factors CLOCK and CYCLE, thereby inhibiting their own transcription (Rutila et al., 1998; Darlington et al., 1998; Yuan et al., 2007; Gentile et al., 2009; Peschel and Helfrich-Förster, 2011; Rund et al., 2011). Photoperiod regulates the clock through CRYPTOCHROME-1 (CRY1), which binds to and degrades TIM in the presence of light (Yuan et al., 2007; Peschel and Helfrich-Förster, 2011; Helfrich-Förster, 2020). Beyond maintaining daily rhythms, the circadian clock regulates a wide range of physiological and behavioural processes, including key mosquito traits such as flight activity and host-seeking (Keegan et al., 2007; Gentile et al., 2009; Duffield, 2024). Changes in the expression of circadian clock genes has been associated with dormancy, suggesting a mechanistic link between seasonal plasticity and clock regulation (Meuti et al., 2015; Huang et al., 2015; Chang and Meuti, 2020).

In this study, we investigated the effects of photoperiod and temperature on thermal plasticity and dormancy-related traits in adult *Ae. aegypti* females. Cold and warm acclimation was attempted under two distinct photoperiods: a short-day (SD) photoperiod representing a standard tropical photoperiod and a long-day (LD) photoperiod, representing a high-summer photoperiod expected to be well beyond the critical photoperiod. We hypothesized that photoperiod interacts with acclimation temperature to shape thermal plasticity in *Ae. aegypti*. Specifically, we predicted that if we exposed mosquitoes to the LD photoperiod then it would constrain thermal plasticity, resulting in reduced low-temperature tolerance. The LD photoperiod was introduced at three different life stages: in the parental generation, in the egg stage and only during adult acclimation to explore life-stage-specific or parental photoperiodic programming of thermal plasticity. Based on the established role of the circadian clock in photoperiodism, two circadian master regulators, *period* and *timeless*, were profiled under both photoperiods and acclimation temperatures. Finally, the effects of temperature and photoperiod on reproductive dormancy-related traits were explored through blood-feeding behaviour assays and the transcript abundance of diapause-implicated genes. Our findings indicate that *Ae. aegypti* exhibits a photosensitive phenotype that reflects a complex seasonal phenotype that includes greater cold tolerance and reproductive quiescence.

## MATERIALS AND METHODS

### Animals

**Fig. 1.**
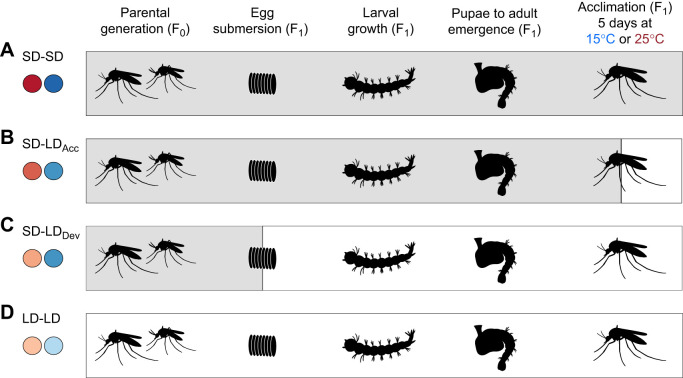
**Outline of *Aedes aegypti* treatment rearing and treatment conditions.** Parental generations and all developmental stages were initially maintained at 28°C until moved to their respective acclimation temperatures as adults. Photoperiod conditions are defined as either short day (SD; 12 h light:12 h dark) or long day (LD; 20 h light:4 h dark), represented in the figures by grey and white backgrounds, respectively. Each thermal acclimation treatment group within each photoperiodic exposure group is denoted by a unique colour-coded circle beneath its label in subsequent figures, red (warm-acclimated) and blue (cold-acclimated).

Two laboratory colonies kept at different photoperiods were established from a shared pool of eggs from an *Aedes aegypti* Linnaeus 1762 colony kept at the University of Florida. This pool originated from a wild population collected in Lee County, FL in 2017 with periodic refreshment of wild material (Carvalho et al., 2022). Experimental colonies were reared as previously outlined (De Nicola et al., 2025). Briefly, egg sheets were submerged in rearing bins containing a nutrient slurry that was prepared by adding 0.05 g of Brewer's yeast and 0.25 g of Tetra^®^ TetraMin^®^ Tropical Flakes (Tetra, Blacksburg, VA, USA) to 700 ml of dechlorinated distilled water (Tetra^®^ AquaSafe^®^ Water Conditioner) and stirred over night at room temperature. Rearing bins (31 cm long×18 cm wide ×11.5 cm high) were maintained at 1 litre volume and larvae were supplemented with 0.25 g Tetra^®^ TetraMin Tropical Flakes whenever there appeared to be no food in the pan, until pupation. Once they reached adulthood, the mosquitoes were provided with unlimited access to dechlorinated distilled water and a 10% sucrose solution. For egg production, bovine blood sourced from a local abattoir was provided through an artificial membrane (Parafilm) and a 15 ml tube of CO_2_ gas sealed with Parafilm to encourage feeding. The short-day (SD) colony was maintained with a light cycle of 12 h light:12 h dark and the long-day (LD) colony with a light cycle of 20 h light:4 h dark with both kept at 28°C (Fig. 1). Treatment groups were established using eggs from the SD and LD colonies (Fig. 1).

Female pupae were identified and collected by assessing the size of the genital lobe under a dissection microscope (Carvalho et al., 2014; Moreno et al., 2021). Collected females were randomly assigned to treatment groups and acclimation temperatures. Two groups, SD-SD and LD-LD remained at their respective light cycles for the duration of their development and acclimation treatments (Fig. 1A,D). The other two groups, SD-LD_Acc_ and SD-LD_Dev_, originated from eggs laid by the SD colony, but their photoperiod was switched to LD either at adult emergence (SD-LD_Acc_; Fig. 1B) or at the time of egg submersion, such that larval and pupal development occurred under LD conditions (SD-LD_Dev_; Fig. 1C). For all photoperiod treatment groups, adults emerged at 28°C with unlimited access to a 10% sucrose solution. At 2 days post adult emergence, female mosquitoes of each group were moved to incubators set to either 15°C (cold) or 25°C (warm) acclimation temperatures and light cycle for 5 days (Jass et al., 2019; De Nicola et al., 2025).

### Chill coma onset assay (CCO)

After 5 days at their adult acclimation temperature, females were aspirated into individual 7 ml glass vials that were affixed to a metal rack and submerged into a custom-built glass tank containing a mixture (1:1 v/v) water and ethylene glycol at 25°C (Davis et al., 2021; O'Neill et al., 2021). The temperature in the tank was held at 25°C for 5 min and then gradually lowered by 0.13°C min^−1^ following Jass et al. (2019). Chill coma onset assay (CCO) temperature was recorded as the temperature at which the insect became completely unresponsive to mechanical stimulus (tapping of the glass vials) and entered a state of complete neuromuscular paralysis where no further movement was observed (MacMillan and Sinclair, 2011; Hazell and Bale, 2011; Overgaard and MacMillan, 2017; Andersen and Overgaard, 2019; Jass et al., 2019). The assay was repeated three to four times per treatment group across three different cohorts with a total of *n*=10–15 female mosquitoes per acclimation temperature and photoperiod treatment.

### Low-temperature survival

To evaluate survival following a low-temperature exposure, cold- and warm-acclimated SD-SD and LD-LD treatment groups were exposed to 0°C for 6 h. These temperatures and durations were selected based on LT_50_ results reported by Jass et al. (2019). Following the cold acclimation treatment outlined above, adult females (*n*=7–10 per cage, 5–6 cages per treatment) were aspirated into plastic vials and submerged in a cooling bath containing a pre-chilled mixture of water and ethylene glycol maintained at 0°C. Following the 6 h exposure, mosquitoes were returned to their cages at respective acclimation temperatures, provided with 10% sucrose solution, to recover. Survival was recorded at 24 and 48 h post cold exposure, with individuals classified as alive if they could stand, walk and/or fly, and dead if they were unresponsive to mechanical disturbance or responsive but unable to right themselves (Rinehart et al., 2010; Jass et al., 2019). Mosquitoes were excluded from the dataset if there was evidence of leakage in their vial (fluid from the tank entered the vial through a broken seal).

### Circadian clock profiling

Circadian clock profiling was performed on the two treatment groups maintained at constant light cycles, SD-SD and LD-LD and following the same thermal acclimation protocol described above. On the fifth day of acclimation, four mosquitoes were collected every 4 h over a 24 h period, starting 2 h after lights on. They were collected individually in 1.5 ml microcentrifuge tubes, flash-frozen in liquid nitrogen, and stored at −80°C. Total RNA was extracted from each individual mosquito following a modified protocol by Chomczynski and Mackey (1995). Briefly, flash frozen samples were homogenized in 0.5 ml TRI Reagent^®^ (Molecular Research Centre, Cincinnati, OH, USA) and incubated on ice for 5 min. The homogenate was centrifuged at 10,000 ***g*** for 5 min at 4°C. The supernatant was transferred to a clean 1.5 ml microcentrifuge tube containing 0.1 ml chloroform on ice and vortexed for 15 s, followed by centrifugation at the same conditions for 15 min. Total RNA was precipitated by removing the clear, aqueous supernatant into 0.25 ml chilled molecular-grade 95% isopropanol and incubated on ice for 10 min. The samples were centrifuged again for 15 min at the same conditions and then washed twice with 70% ethanol. The samples were centrifuged at 7500 ***g*** for 5 min at 4°C between each ethanol wash. The pellet was air dried for 5–10 min at room temperature and resuspended into 25 μl nuclease-free sterile water. RNA quantity, quality and integrity were assessed by the 260/280 nm absorbance ratio (>1.8) using spectrophotometry (Take3 Microvolume Plate, Agilent BioTek, Santa Clara, CA, USA) and gel electrophoresis [1% agarose Tris-acetate-EDTA (TAE; w/v) stained with 1× SYBR™ Safe DNA Gel Stain (Invitrogen, Carlsbad, CA, USA)]. Samples were accepted for analysis if the 260/280 nm absorbance ratio ranged between 1.9 and 2.1, and the agarose gel electrophoresis showed no evidence of nucleic acid degradation. All samples passed these criteria.

Complementary DNA (cDNA) was synthesized using the iScript™ Reverse Transcription Supermix for RT-qPCR (BioRad, Hercules, CA, USA) following the manufacturer's recommendations. Reactions were prepared with 4 μl of 5× iScript RT Supermix (BioRad, Hercules, CA, USA) and 1 μg total RNA to a total volume of 20 μl with nuclease-free, sterile H_2_O. The reaction proceeded for 5 min at 25°C, followed by 20 min at 46°C and the reaction was terminated by 1 min at 95°C. The cDNA was diluted tenfold and stored at −20°C. The relative levels of *period*, *timeless*, *ribosomal protein* (*rp49*) and *actin* were measured using RT-qPCR on CFX Duet Real-Time PCR System (BioRad, Hercules, CA, USA) using primers previously described for circadian clock profiling in *Ae. aegypti* (Gentile et al., 2009) or designed for their target gene (Table S1). Two different reference genes were required for the different acclimation treatments: *rp49* for warm-acclimated samples and *actin* for cold-acclimated samples. This was necessary because acclimation temperature altered transcript abundance of the reference genes under different conditions, from being constant throughout the day to showing cyclical variation. To determine if reference transcript abundance was constant or rhythmic, the raw cycle threshold (*C*_t_) values for each reference gene were fitted to two nonlinear least-square models: a null model that assumed constant abundance and a cosine model that represented cyclical variation over time. The two models were compared using ANOVA.

We generated four biological replicates with each replicate consisting of an individual mosquito for each time point and each acclimation treatment. The RT-qPCR reactions were prepared using 1 μl cDNA, diluted to 100 ng μl^−1^, combined with 5 μl SsoAdvanced Universal SYBR Green Supermix (BioRad, Hercules, CA, USA), 0.125 μl of 20 μmol l^−1^ gene-specific forward and reverse primers (Table S1) and were then brought up to a total volume of 10 μl with nuclease-free, sterile water. Reactions were incubated in a 96-well plate for 30 s at 95°C followed by 35 cycles of 95°C for 10 s and 60°C for 15 s. A melt curve analysis was performed after the cycling for each reaction, and two technical replicates were performed per biological replicate. *C*_t_ values for each biological replicate were calculated as the mean of two technical replicates. Technical consistency was assessed using a 10% variation threshold, and all replicate pairs met this criterion, passing initial quality control.

Relative mRNA abundance was calculated using the 2(−ΔΔ*C*_t_) method. For each biological replicate, the average *C*_t_ value of the target gene was determined and normalized by subtracting the corresponding average *C*_t_ value of the reference gene yielding Δ*C*_t_ values. The time point with the lowest mean Δ*C*_t_ across biological replicates, representing peak transcript abundance, was designated as the reference for normalization. ΔΔ*C*_t_ values were calculated by comparing each time point to this reference, and relative mRNA abundance was expressed as 2^−ΔΔ*C*t^.

### Blood feeding assay

SD-SD and LD-LD males and females were collected at pupation (*n*=15–25 per sex) and warm- or cold-acclimated for 5 days following the protocol described above. Blood-feeding trials were conducted on cold- and warm-acclimated mosquitoes on the fifth day of acclimation at 15°C. Additionally, blood-feeding was attempted after the cages were transferred to 25°C for 1, 3, 24 or 72 h to facilitate rewarming. Warm-acclimated groups were maintained at 25°C for the same duration to represent complementary controls.

Aliquots of frozen bovine blood were heated to 37°C and cages were provided with 5 ml blood in a clean 50 ml tube with an artificial membrane (parafilm) and a 15 ml tube of CO_2_ gas sealed with parafilm to encourage feeding. Access to the 10% sucrose was removed 24 h before blood feeding. After 20 min, the blood was removed, and mosquitoes were frozen at −20°C for later visual inspection of feeding. Females were separated from males, and the numbers of non-fed and blood-fed females were counted. The amount of blood ingested was not documented, with partial and complete engorgement both considered blood-fed.

### Differential transcript abundance of genes implicated in dormancy in other insects

Relative mRNA abundance was evaluated on four biological replicates, each replicate from a single female mosquito, between warm- and cold-acclimated SD-SD female mosquitoes using the 2(−ΔΔ*C*_t_) method. *Aedes aegypti*-specific primers were designed for *FOXO*, *ILP-1* and *allatotropin* (Table S1). Total RNA was extracted and cDNA synthesized following the protocol described above and relative levels of these genes were measured using RT-qPCR as described above. As for circadian clock profiling, *rp49* was used as a reference gene (Gentile et al., 2009). *Rp49* was selected as the reference gene for differential transcript abundance analysis between cold- and warm-acclimated treatment groups at a single time point. This gene has shown relatively stable transcript abundance levels between cold- and warm-acclimated insects, compared with other common reference genes such as *GAPDH* (Enriquez and Colinet, 2019) and is widely used as a reference gene for *Ae. aegypti* and other mosquito species (Gentile et al., 2009, 2013; Chang and Meuti, 2020; Cruz et al., 2023; Wolkoff et al., 2025; De Nicola et al., 2025). A melt curve analysis was performed after the cycling for each reaction, and two technical replicates were performed per biological replicate. The *C*_t_ of each biological replicate was averaged across technical replicates. Technical consistency was assessed using a 10% variation threshold, and all replicate pairs met this criterion, passing initial quality control.

Briefly, the average threshold *C*_t_ for each biological replicate was calculated between technical replicates and normalized by subtracting the average *C*_t_ value of the reference gene *rp49* to calculate the Δ*C*_t_ values. The average Δ*C*_t_ value from the control group was then subtracted from each of the biological replicates Δ*C*_t_ value to calculate ΔΔ*C*_t_. Mean fold change values±s.e.m. were calculated for the warm- and cold-acclimated treatments.

### Data analysis

Data analyses were conducted using R version 4.4.1 (r-project.org). Treatment-specific outliers in the CCO dataset were identified using the rstatix package (https://CRAN.R-project.org/package=rstatix), with outlier coefficient set to 2. A total of seven individuals were removed from the dataset of 274. Normality of the datasets was assessed using a Shapiro–Wilks tests prior to statistical analysis. All datasets met the assumptions of normality (*P*>0.05), so parametric statistical testing was used for downstream analysis. A linear-mixed effects model was used to assess the effects of acclimation temperature, photoperiod, and their interaction on CCO temperature across the entire dataset, with acclimation temperature and photoperiod as fixed effects and cohort as a random effect using the nlme package in R (https://CRAN.R-project.org/package=nlme; Pinheiro and Bates, 2000). Estimated marginal means were obtained using the emmeans package in R (https://CRAN.R-project.org/package=emmeans; Searle et al., 1980).

A generalized linear mixed-effects model (binomial distribution) was used to assess the effects of acclimation temperature, photoperiod and time of assessment on survival as fixed effects, with cage as a random effect. Blood feeding propensity was analysed using a mixed-effects model with acclimation temperature, photoperiod and time warmed (in h) as fixed effects. Model selection for both survival and blood-feeding analyses was performed using Akaike's Information Criterion (AIC). The lme4 package was used to run both models (https://CRAN.R-project.org/package=lme4; Bates et al., 2015).

For circadian clock gene profiling, outlier detection was performed as described above for each treatment independently at each timepoint based on their Δ*C*_t_ value. A total of seven and nine individual data points for *timeless* and *period* were excluded, respectively (see Table S3 for outliers removed for circadian clock genes). To assess rhythmicity of *period* and *timeless* transcript levels, relative fold change at each timepoint was fit to a cosine function using nonlinear least squares regression (nls) using R version 4.4.1 (r-project.org). The model followed: Relative fold change∼*m*+*A*cos(ω*T*+Φ), where *m* is the mesor (mean abundance), *A* is the amplitude, *T* is time in hours, Φ is the phase-shift and ω=2π/24, corresponding to the angular frequency of a 24 h period (Refinetti et al., 2007). Model fit was visualized by generating predicted values over 24 h and plotted alongside observed data represented by mean relative fold change ±s.e.m. at each time point. Goodness of fit was evaluated by calculating *R*^2^ values. The cosine model was then compared with a null model assuming constant transcript abundance over time (Relative fold change∼*m*) using an ANOVA on nested nls fits to determine if rhythmicity was statistically supported. A linear mixed-effects model: Relative fold change∼Photoperiod×cos*T*+sin*T* was used to assess whether photoperiod influenced the amplitude and timing of period and timeless transcript profiles in treatment groups were rhythmic patterns were detected (Refinetti et al., 2007). For the dormancy-implicated genes, significance was tested using a two-sample *t-*test on log-transformed fold-change values with *P*≤0.05 indicating significant changes compared with the control (warm-acclimated).

## RESULTS

### Chill coma onset temperature

**Fig. 2.**
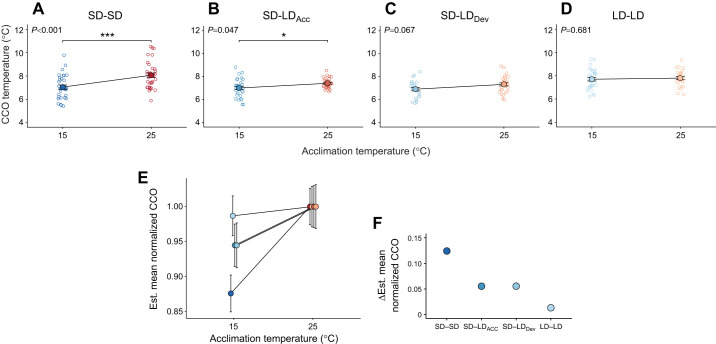
**Chill coma onset (CCO) temperatures across acclimation treatments.** (A–D) CCO temperature for cold-acclimated (blue) and warm-acclimated (red) groups in the four treatment conditions: SD-SD, SD-LD_Acc_, SD-LD_Dev_ and LD-LD, respectively (*n*=25–35 per treatment group, 3–4 assay replicates). Pairwise comparisons for each group were tested using a *post hoc* Tukey test. (E) Mean normalized CCO values for each treatment condition. (F) ΔEst. mean normalized CCO values for each treatment group. Greater values of ΔEst. reflect a greater degree of thermal plasticity in CCO. ***P<0.001, *P<0.05.

Both acclimation temperature and photoperiod treatment had significant main effects on CCO temperature, and their interaction was also significant (Table 1). The normalized estimated mean CCO temperature for each group was calculated by normalizing each group to the estimated mean of their warm-acclimated treatment. There was a clear decrease in CCO temperature in the SD-SD 15°C acclimation group (Fig. 2A), but there was no acclimation effect detectable in the LD-LD 15°C acclimation group (Fig. 2D, Table 1). There was a trend towards acclimation at 15°C lowering CCO temperature in both the SD-LD_Acc_ (Fig. 2B) and SD-LD_Dev_ (Fig. 2C) treatment groups, but this trend only reached our threshold value for significance (*P*<0.05) in the SD-LD_Acc_ group and not in the SD-LD_Dev_ group.

**Table 1.** Summary of linear mixed-effects model assessing the effects of photoperiod and acclimation on chill coma onset (CCO) temperature

Effect	χ^2^	d.f.	*P*-value
**Acclimation**	25.63	1	<0.001***
**Photoperiod**	19.04	3	<0.001***
**Acclimation×Photoperiod**	10.33	3	0.012*

Significant effects are highlighted in bold.

*Post hoc* Tukey-adjusted comparisons based on the estimated marginal means revealed distinct photoperiod responses within each acclimation group (Fig. S1). The LD-LD cold-acclimated group had a significantly higher estimated mean CCO temperature compared with the group maintained under SD conditions (*P*<0.05) as well as both SD-LD_Dev_ (*P*<0.01) and SD-LD_Acc_ (*P*<0.01) groups. In contrast, in the warm-acclimated groups, estimated mean CCO temperature was significantly higher under SD-SD conditions, relative to both SD-LD_Dev_ (*P*<0.01) and SD-LD_Acc_ (*P*<0.01). However, for the warm-acclimated groups, no significant differences were detected between the LD-LD group and any of the other photoperiod treatments.

The normalized estimated mean CCO temperature for each group was calculated by normalizing each group to its estimated mean for the warm-acclimated treatment. The largest change in normalized CCO temperature in response to cold acclimation (an index of the degree of thermal plasticity) was observed in the SD-SD group with this value gradually decreasing across the other groups (Fig. 2F). Given the pronounced difference in acclimation response between these two acclimation groups, we focused the remainder of the study on the two treatment groups that were maintained under consistent photoperiods: SD-SD and LD-LD.

### Survival after cold stress

**Fig. 3.**
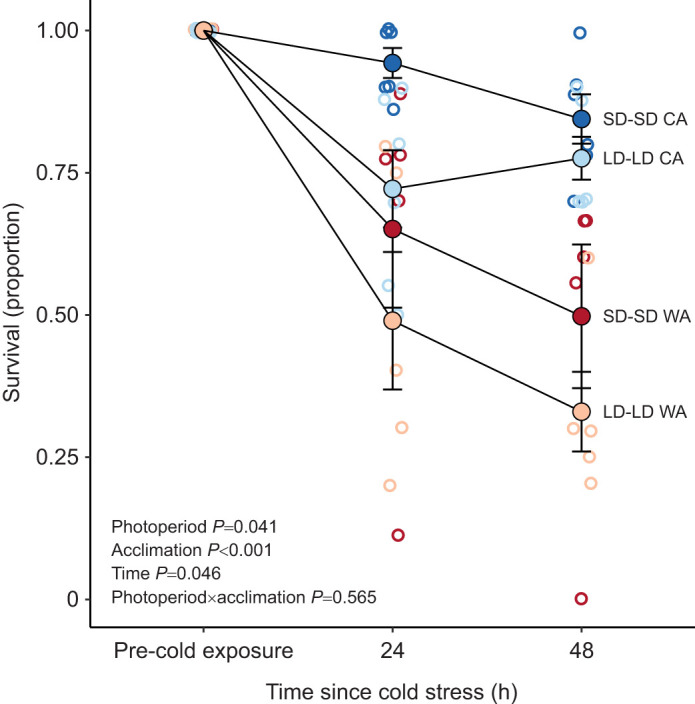
**Survival proportion per cage of warm- (WA) and cold-acclimated (CA) SD-SD and LD-LD treatment groups following exposure to 0°C for 6 h.** Survival was assessed 24 h and 48 h following cold exposure. Each individual open circle represents a replicate cage (*n*=7–10 adult females per cage, 5–6 replicate cages per treatment group). Solid circles represent the mean (±s.e.m.) survival per treatment group. Summary of generalized linear mixed-effects model assessing the effects of photoperiod, acclimation and time, reduced using Akaike's Information Criterion (AIC).

Photoperiod, acclimation and time have significant main effects on survival post cold stress; however, no significant interactions were detected (Fig. 3). The cold-acclimated treatment groups had a higher proportion of females alive after a cold exposure than warm-acclimated groups, but there was no difference in survival between the SD-SD and LD-LD cold-acclimated groups 48 h after cold exposure. A decrease in survival was seen between 24 h and 48 h in all groups except the LD-LD cold-acclimated group (Fig. 3).

### Circadian clock gene profiling

**Fig. 4.**
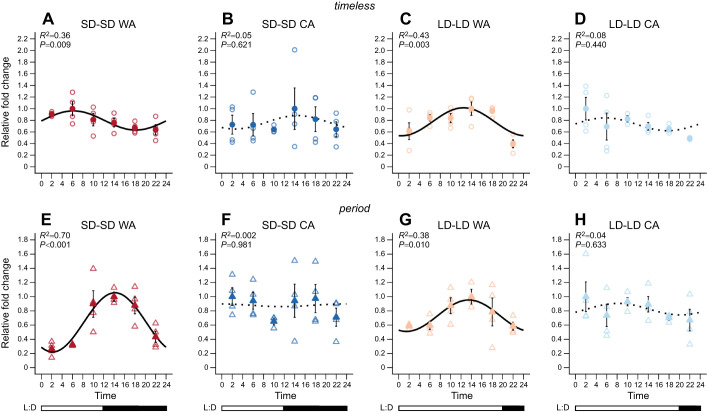
**Temporal transcript abundance profiles of *timeless* and *period* in warm- and cold-acclimated *Ae. aegypti* females.** Gene expression of *timeless* (A–D) and *period* (E–H) was measured using quantitative PCR in warm-acclimated and cold-acclimated SD-SD and LD-LD mosquitoes over 24 h (*n*=3–4 individual mosquitoes per time point, two technical replicates per sample). Filled circles and triangles represent mean transcript abundance values (±s.e.m.) and open circles and triangles represent values from individual mosquitoes. A cosine function was fitted to each profile. Profiles with significant fit (*P*<0.05) are shown as solid lines, whereas non-significant fits are shown as by dotted lines. The coefficient of determination (*R*²) indicates goodness of fit of the cosine function.

Acclimation temperature (15°C) affected the rhythmicity of circadian clock genes *timeless* and *period* (Fig. 4). For *timeless*, statistically significant rhythmicity was only present in the warm-acclimated treatment groups for both SD-SD and LD-LD (Fig. 4A; *R*^2^=0.36, *P*=0.009, Fig. 4C; *R*^2^=0.43, *P*=0.003, respectively), and there was no clear rhythmicity in the groups exposed to the cold-acclimation treatment, SD-SD and LD-LD (Fig. 4B, *R*^2^=0.05, *P*=0.621, Fig. 4D; *R*^2^=0.08, *P*=0.44, respectively). Similar results were observed for *period* transcript abundance, where the only treatment groups to display evidence for rhythmicity were warm-acclimated SD-SD and LD-LD (Fig. 4E; *R*^2^=0.70, *P*<0.001, Fig. 4G; *R*^2^=0.38, *P*=0.010, respectively). No evidence for *period* rhythmicity was present in the SD-SD and LD-LD groups exposed to the cold-acclimation treatment (Fig. 4F; *R*^2^=0.002, *P*=0.981, Fig. 4H; *R*^2^=0.04 *P*=0.633, respectively).

Rhythmic transcript abundance of *period* and *timeless* was observed in the warm-acclimated treatment groups for both photoperiods (Fig. 4); thus, a fixed-effects model was used to determine whether the rhythms of these circadian clock genes differed when females were reared under different photoperiods. Photoperiod did not have a significant main effect on the relative transcript abundance of *period* or *timeless* (*P*=0.137 and *P*=0.675 respectively, Tables S2 and S3). For *timeless*, there were, significant interactions in the effects of photoperiod and both cos*T* (Table S2, *p*=0.004) and sin*T* (Table S2, *p*=0.005) on transcript levels. *Timeless* relative transcript abundance peaked approximately 6 h later in the LD-LD group, compared with the SD-SD group (Fig. 4A,C). This suggests that the long photoperiod changed both the timing (phase) and amplitude (shape) of the rhythm of *timeless* expression. No such significant interactions were detected for *period* transcript abundance (Table S3).

### Blood-feeding

**Fig. 5.**
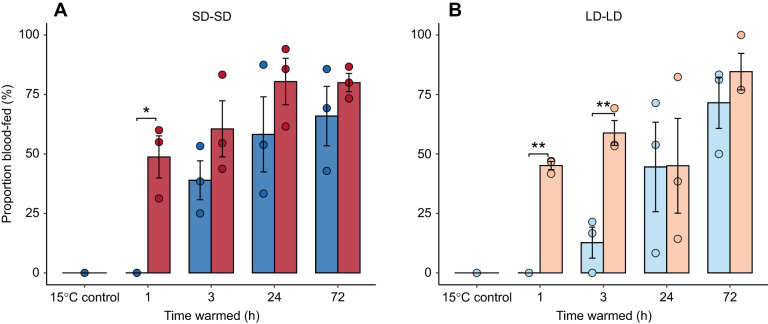
**Effect of temperature and photoperiod on blood-feeding behaviour in *Ae. aegypti*.** Proportion of cold- and warm-acclimated mosquitoes from the SD-SD (A) and LD-LD (B) groups and following rewarming to 25°C. Feeding at 15°C was included as a negative control following Jass et al. (2019). Bars represent mean±s.e.m. of three biological replicates (open circles; *n*=11–22 males and females per biological replicate). **P<0.01, *P<0.05, two sample *t*-test.

**Fig. 6.**
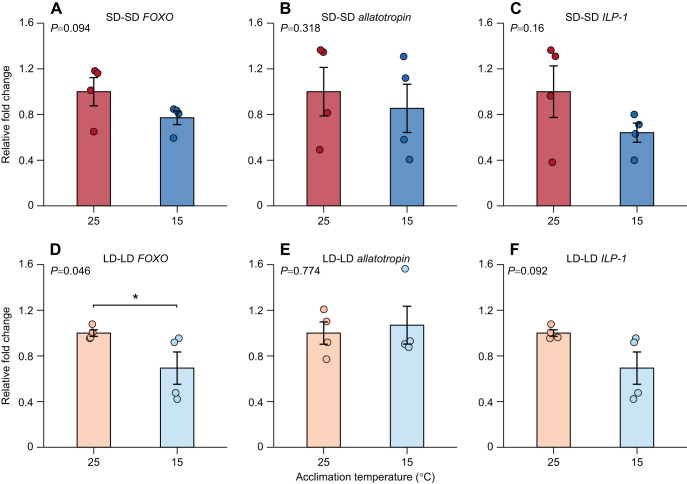
**Relative transcript abundance levels of *FOXO*, *allatotropin* and *ILP-1* in warm- and cold-acclimated SD-SD and LD-LD females.** *FOXO* (A,D), *allatotropin* (B,E) and *ILP-1* (C,F) transcript levels shown as means±s.e.m. of four biological replicates (*n*=1 mosquito per biological replicate, two technical replicates per sample). *P<0.05, two-sample *t*-test.

Acclimation temperature and time warmed had significant effects on the percentage of females that blood fed (Table 2). Like findings reported by Jass et al. (2019), cold-acclimated mosquitoes at both photoperiods did not blood feed at 15°C (Fig. 5, 15°C control). After 1 h of rewarming at 25°C, cold-acclimated females from both photoperiods remained reluctant to blood feed (Fig. 5). A statistically significant difference in proportion of blood-fed females was observed between warm- and cold-acclimated females (Fig. 6A,B; *P*=0.032 for SD-SD and *p*=0.001 for LD-LD, two-sample *t*-test). The SD-SD cold-acclimated group resumed blood feeding at a proportion comparable to the warm-acclimated control group after 3 h of rewarming (Fig. 5A, *P*=0.21, two-sample *t*-test). In contrast, the LD-LD cold-acclimated group took 24 h to reach a feeding proportion that was not significantly different from the warm-acclimated control (Fig. 5B, *P*=0.006 at 3 h of rewarming and *p*=0.986 at 24 h of rewarming, two-sample *t*-test).

**Table 2.** Summary of linear mixed-effects model assessing the effects of photoperiod, acclimation and number of hours warmed on proportion of females that blood fed, reduced using Akaike's Information Criterion (AIC)

Effect	χ^2^	d.f.	*P*-value
**Acclimation**	15,170.7	1	<0.001***
Photoperiod	828.2	1	0.105
**Time warmed**	23,874.3	4	<0.001***
**Time warmed×Acclimation**	2604.4	3	0.048*

### Transcript abundance of dormancy-implicated genes

Cold acclimation did not significantly impact the transcript abundance of *ILP-1* or *allatotropin* in the SD-SD or LD-LD treatment groups (Fig. 6B,C,E,F). Contrary to expectations for a diapause phenotype, *FOXO* was significantly reduced in the LD-LD cold-acclimated group. Although the SD-SD group showed a similar trend, it did not reach statistical significance (Fig. 6A,D).

## DISCUSSION

This study presents the first evidence for a photosensitive adult seasonal cold-tolerance phenotype in *Ae. aegypti* that is dependent on the day length experienced early in development. Cold-acclimated *Ae. aegypti* females exhibited several key characteristics commonly associated with mosquito dormancy, such as enhanced low temperature tolerance and developmental arrest (Denlinger and Armbruster, 2014, 2016). Prior to this study, a quiescence phenotype had been described only in the egg, embryonic and pharate larval stages of *Ae. aegypti* (Sota and Mogi, 1992; da Silva and da Silva, 1999; Rezende et al., 2008; McMeniman and O'Neill, 2010; Perez and Noriega, 2012, 2013; Vargas et al., 2014; Prasad et al., 2023).

In this study, exposure to a long-day photoperiod modulated the capacity of *Ae. aegypti* to cold acclimate, confirming the photosensitive nature of this phenotype. This finding is consistent with previous work demonstrating the photoperiodic regulation of cold tolerance across other insect species (Lanciani et al., 1992; Rodgers et al., 2006; Vesala and Hoikkala, 2011; Fischer et al., 2012; Bauerfeind et al., 2014; Manenti et al., 2021; Sapre et al., 2025), and it is well documented that photoperiod regulates seasonal responses of many insect species, including mosquitoes (Denlinger and Armbruster, 2014; Saunders, 2020; Goto, 2022). While demonstrating the photosensitivity of the adult cold-acclimation phenotype, we also show that the magnitude of this plasticity depends on the life stage at which the LD photoperiod was introduced. Thermal plasticity was greatest when mosquitoes were maintained under SD conditions throughout development, whereas exposure to the LD photoperiod reduced the ability to cold acclimate. This reduction was most pronounced when the LD photoperiod was experienced across generations, suggesting that early-life or maternal photoperiodic cues may shape adult thermal responses.

The lack of thermal plasticity observed in the LD-LD group, compared with the SD-SD group, suggests that maternal photoperiod influences thermal plasticity and shapes offspring responses in *Ae. aegypti*, which is consistent with a maternal effect. Previous studies on both *Aedes albopictus* and *Ae. Aegypti*, have shown that maternal short-day photoperiods (8–10 h of light in a 24 h period) enhance stress-resistant traits in eggs, such as larger size, higher triglyceride content and increased volume compared with long day photoperiod (14–16 h of light in a 24 h period) (Lacour et al., 2014; Fischer et al., 2019; Mensch et al., 2021; Lee et al., 2024). Short-day photoperiods also shorten offspring development time and wing length in *Ae. aegypti* reared at 23°C (Loetti et al., 2021). However, a photoperiodic effect on stress resistance during later life stages, such as adulthood, had not been demonstrated until now. That said, it is important to note that our colonies were reared at 28°C prior to cold acclimation. This high temperature rearing may have constrained the expression of a stronger dormancy phenotype under SD conditions because higher temperatures have been shown to supress photoperiodic dormancy responses in other insect species (Pumpuni et al., 1992; Doležal and Sehnal, 2007).

Cold acclimation (15°C) improved survival following cold stress in both photoperiods, with the SD-SD groups outperforming their LD-LD counterparts (Fig. 3). Assessment time had a significant main effect on survival post- cold-stress (Fig. 3), with a decline in all groups from 24 h to 48 h post cold exposure except for the LD-LD cold-acclimated group. This decline in survival between 24 to 48 h after cold stress may reflect latent injury, or delayed chilling injury caused by the prolonged cold stress, a phenomenon previously reported in female fruit flies (Colinet et al., 2017; Ritchie et al., 2021; Allen et al., 2024; El-Saadi et al., 2025).

A significant interaction between photoperiod and acclimation was observed for CCO temperature, but not for survival after cold stress. Cold acclimation improved survival after a cold stress, indicating largely additive effects of photoperiod and acclimation temperatures on cold stress survival. In contrast, patterns for CCO were markedly different. Pairwise comparisons within acclimation groups showed that photoperiod-dependent differences in CCO were expressed in an acclimation-specific manner, with cold acclimation evident only under short-day conditions, particularly in the SD-SD cold-acclimated group. This indicates a non-additive interaction in which photoperiod strongly modulates the effects of cold acclimation on chill susceptibility. This outcome is not surprising because the physiological mechanisms underlying these traits differ (Davis et al., 2021). CCO is a state of complete neuromuscular paralysis that occurs because of loss of central nervous system function (Armstrong et al., 2012; Andersen et al., 2018). The physiological causes underlying chill injury after cold stress are, however, more complex as multiple mechanisms have been proposed based on the intensity and duration of the exposure (Sinclair and Roberts, 2005). A well-supported mechanism behind chill injury is the progressive disruption of hemolymph ionic and osmotic homeostasis, resulting in hemolymph hyperkalemia that causes cell depolarization and ultimately cell death (MacMillan et al., 2015a; MacMillan, 2019; Bayley et al., 2019).

In addition to enhanced low temperature tolerance, cold acclimation in adult *Ae. aegypti* also appears to suppress cyclical transcript abundance of two key circadian clock regulators, *timeless* and *period*. The profiles of circadian clock genes before, during and after diapause have been extensively studied in *Cx. pipiens* (Meuti and Denlinger, 2013; Meuti et al., 2015; Chang and Meuti, 2020; Wolkoff et al., 2025). Many circadian clock genes and their targets will continue to cycle throughout diapause and after diapause in *Cx. pipiens* (Meuti et al., 2015; Chang and Meuti, 2020). The silencing of key clock genes such as *cycle*, *clock* and *period* has been shown to effectively disrupt the normal physiological response to photoperiod, preventing the typical expression of diapause-related species in several insect species (Meuti et al., 2015; Zhu et al., 2019; Iiams et al., 2019; Chang and Meuti, 2020; Goto and Nagata, 2022). Changes in core circadian clock gene transcript abundance have been observed during cold acclimation in various species of fruit fly (Vesala et al., 2012; Poikela et al., 2021). However, to our knowledge, the effect of cold acclimation on rhythmic transcript abundance of core clock genes has not yet been studied.

Under warm conditions, *timeless* and *period* exhibited rhythmic relative transcript abundance patterns consistent with prior studies in *Ae. aegypti* (Gentile et al., 2009). However, the LD photoperiod altered the phase of *timeless* transcript abundance so that it peaked roughly 6 h later than in the SD photoperiod. Of the two circadian clock genes profiled in this study, *timeless* is considered more light sensitive, as it is directly regulated by light via CRY1, which binds TIM and degrades it in the presence of light (Yuan et al., 2007; Peschel and Helfrich-Förster, 2011; Helfrich-Förster, 2020). The reshaping of *timeless* relative transcript abundance we observed could therefore result from changes in light-sensitive interactions, such as CRY1–TIM association, under LD photoperiods.

Under cold acclimation conditions, transcript abundance of both clock genes remained at a relatively constant level with no detectable cycling. The circadian clock of *Ae. aegypti* is known to be sensitive to both temperature and photoperiod (Rivas et al., 2018; Teles-de-Freitas et al., 2020; Cruz et al., 2023), but prior studies largely focused on higher temperatures than those used here (between 20°C and 30°C). Rhythmic expression of *timeless* was previously abolished when daily rhythms of photoperiod and temperature gave conflicting signals (Teles-de-Freitas et al., 2020). There are no other studies, to our knowledge, that show low temperatures abolishing the rhythmicity of core circadian clock genes in insects, but rather that they alter the phase or amplitude of clock genes in *D. melanogaster* and another mosquito species, *Anopheles stephensi* (Boothroyd et al., 2007; Wang et al., 2021).

A functional circadian clock is essential for *Ae. aegypti* activity, host-seeking and mating behaviour, all of which follow diurnal patterns (Gentile et al., 2009, 2013; Lima-Camara et al., 2011; Rund et al., 2020; Liu et al., 2022). After a blood meal, there is a downregulation of core circadian clock genes, *timeless*, *period*, *clock* and *cycle*, in female *Ae. aegypti* (Gentile et al., 2013). Female mosquitoes also exhibit a reduction in host-cue responses after a blood meal, and it has been hypothesized that this is due to the downregulation of circadian clock genes (Meireles-Filho et al., 2006). Although we did not directly compare transcript abundance of *period* and *timeless* between warm- and cold-acclimated mosquitoes, we did see a loss of rhythmic transcript abundance of these two circadian clock genes with cold acclimation.

Consistent with this, mosquitoes maintained at low temperature did not blood feed, but this behaviour was recovered following rewarming. The return to blood feeding after 3 h at 25°C suggests that the antifeedant response to cold in the SD-SD photoperiod group is rapidly reversible, characteristic of quiescence rather than programmed diapause (Denlinger, 2023). The cold-acclimated LD-LD group took longer to recover a blood-feeding behaviour compared with their warm-acclimated counterpart. This slower recovery may indicate reduced tolerance to the low temperature exposure during cold acclimation because of the high summer photoperiod under which they were held. Low temperatures can impair sensory perception, neuromuscular function and metabolic activity, all of which are necessary for blood-feeding behaviour (Overgaard and MacMillan, 2017). The prolonged suppression of blood-feeding in the LD-LD cold-acclimated females may reflect a slower physiological recovery or greater latent injury relative to the SD-SD females.

The reversible reproductive arrest observed in cold-acclimated *Ae. aegypti* does not appear to be controlled by the IS and JH pathways, as would be expected if this phenotype was representative of diapause. If this seasonal phenotype were representative of an adult diapause state, we would expect to see downregulation of *ILP-1* and *allatotropin*, accompanied by upregulation of *FOXO*, as in *Cx. pipiens* (Sim and Denlinger, 2008; Sim et al., 2015). However, no significant differences were detected in the transcript abundance of these genes between SD-SD cold- and warm-acclimated groups (Fig. 6A–C). The only significant change in transcript abundance was a downregulation of *FOXO* in the LD-LD cold-acclimation group, the opposite pattern from what one would expect for a programmed seasonal diapause response, further supporting our conclusion that this seasonal phenotype reflects quiescence rather than diapause.

With the rapid range expansion of this important disease vector into new regions, understanding *Ae. aegypti* thermal plasticity and its ability to persist in temperate environments is critical (Kraemer et al., 2019). In this study, we provide the first evidence of an adult seasonal phenotype in *Ae. aegypti* that is likely to contribute to its survival in unfavourably cold conditions. Given the photosensitive and rapidly reversible nature of this thermal acclimation phenotype, we propose that this is an adult quiescence that can help *Ae. aegypti* persist in cold weather conditions, which had only been described thus far at the egg and embryo stages in this species (Diniz et al., 2017). This seasonal phenotype may help to facilitate this species' range expansion by enabling individuals to withstand periods of cold exposure, particularly in autumn and spring where cold shocks are common in temperate climates. The high thermal plasticity described in this study may be contributing to the species' persistence and continued spread into previously unpopulated temperate regions. Future work will be needed to fully understand how a range of seasonally and geographically relevant photoperiods could affect the persistence and abundance of this disease-vectoring, invasive mosquito in new areas of expansion.

## Supplementary Material

10.1242/jexbio.252460_sup1 Supplementary information

Dataset 1. Experimental data for blood-feeding assay

Dataset 2. Experimental data for chill-coma onset (CCO) assay

Dataset 3. Experimental data for low-temperature exposure survival assay

Dataset 4. Experimental qPCR data for diapause-implicated genes

Dataset 5. Experimental qPCR data for *period* circadian clock gene profiling

Dataset 6. Primer efficiency calculations

Dataset 7. Experimental qPCR data for *timeless* circadian clock gene profiling
