# Supplementary material for: Early photoperiodic cues modulate capacity for adult cold tolerance in the yellow fever mosquito Aedes aegypti
Source: J Exp Biol. 2026 Jun 18;229(12):jeb252460. doi: 10.1242/jeb.252460 (PMC13327540; doi:10.1242/jeb.252460)
Supplement: Supplementary information [file jexbio-229-252460-s1.pdf]

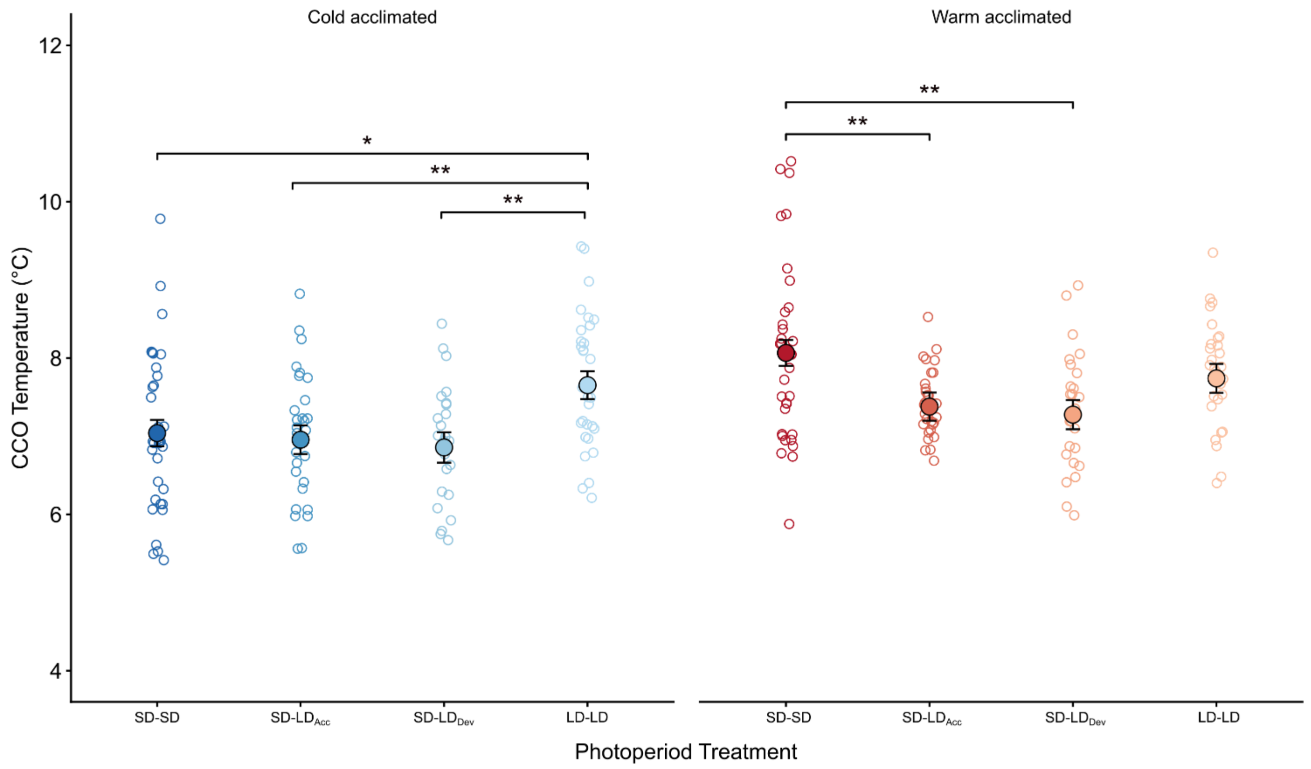

Fig. S1. Estimated mean chill coma onset (CCO) temperature across photoperiod treatment and acclimation temperature. Points represent the estimated marginal means of CCO per photoperiod temperature derived from the linear mixed-effects mode and open circles show individual CCO observations. Cold-acclimated groups are shown in blues (left plot) and warm-acclimated groups in reds (right). Pairwise comparisons for each group were tested using a post-hoc Tukey test. ** $p < 0.01$, * $p < 0.05$

Table S1. Primer sequences for circadian clock profiling and dormancy-implicated genes used for RT-qPCR

Primer Name	Sequence (5'-3')	Accession	Primer Efficiency	R^2	Reference
Allatotropin_fwd	ATGACCGCAAGAGGGTTCGGGA	XM_001660152.2	100.67	0.996	
Allatotropin_rev	GCATTGTCGGCGGTGAGCTCTT	XM_001660152.2			
ILP-1_fwd	GGTTCCTACCGGGAGCAGCTGA	DQ845750.1	109.42	0.999	
ILP-1_rev	AAACTGAAGCGCTTGGGGAGCC	DQ845750.1			
FoxO_fwd	ATGACCGCAAGAGGGTTCGGGA	XM_021852675.1	102.91	0.997	
FoxO_rev	GCATTGTCGGCGGTGAGCTCTT	XM_021852675.1			
Actin_fwd	GCGTCCATCAGGCGTTGTTCGA	XM_001649482.2	103.72	0.993	
Actin_rev	CGTTCACGCATTCCCCCGAACA	XM_001649482.2			
Rp49_fwd	GCTATGACAAGCTTGCCCCCA		95.64	0.997	Gentile *et al.*, 2009
Rp49_rev	TCATCAGCACCTCCAGCTC				
timeless_fwd	ATCGAACAACTTTGGGAGC		111.09	0.996	Gentile *et al.*, 2009
timeless_rev	CGATCCCGTAAAACCTGTATG				
period_fwd	CGTTGAAAAGTCTCAATCAACCT		107.28	0.999	Gentile *et al.*, 2009
period_rev	GCTGAGTGAAAGCTTCGCCG				

Table S2. Summary of linear mixed-effects model assessing the effects of photoperiod on *timeless* transcript abundance

Effect	X^2	Df	p-value
Photoperiod	0.006	1	0.675
Cos hour	0.0007	1	0.887
**Sin hour**	0.325	1	**0.004**
**Photoperiod × cos hour**	0.322	1	**0.005**
**Photoperiod × sin hour**	0.228	1	**0.016**

Journal of Experimental Biology • Supplementary information

Table S3. Summary of linear mixed-effects model assessing the effects of photoperiod on *period* transcript abundance

Effect	X^2	Df	p-value
Photoperiod	0.095	1	0.137
**Cos hour**	1.374	1	**< 0.001**
**Sin hour**	0.544	1	**0.001**
Photoperiod × cos hour	0.115	1	0.105
Photoperiod × sin hour	0.110	1	0.113

Dataset 1. Experimental data for blood-feeding assay

Available for download at
https://journals.biologists.com/jeb/article-lookup/doi/10.1242/jeb.252460#supplementary-data

Dataset 2. Experimental data for chill-coma onset (CCO) assay

Available for download at
https://journals.biologists.com/jeb/article-lookup/doi/10.1242/jeb.252460#supplementary-data

Dataset 3. Experimental data for low-temperature exposure survival assay

Available for download at
https://journals.biologists.com/jeb/article-lookup/doi/10.1242/jeb.252460#supplementary-data

Dataset 4. Experimental qPCR data for diapause-implicated genes

Available for download at
https://journals.biologists.com/jeb/article-lookup/doi/10.1242/jeb.252460#supplementary-data

Dataset 5. Experimental qPCR data for *period* circadian clock gene profiling

Available for download at
https://journals.biologists.com/jeb/article-lookup/doi/10.1242/jeb.252460#supplementary-data

Dataset 6. Primer efficiency calculations

Available for download at
https://journals.biologists.com/jeb/article-lookup/doi/10.1242/jeb.252460#supplementary-data

Dataset 7. Experimental qPCR data for *timeless* circadian clock gene profiling

Available for download at
https://journals.biologists.com/jeb/article-lookup/doi/10.1242/jeb.252460#supplementary-data

Journal of Experimental Biology • Supplementary information
